# Two types of peak emotional responses to music: The psychophysiology of chills and tears

**DOI:** 10.1038/srep46063

**Published:** 2017-04-07

**Authors:** Kazuma Mori, Makoto Iwanaga

**Affiliations:** 1 Center for Information and Neural Networks (CiNet), National Institute of Information and Communications Technology, and Osaka University, Suita-shi, Osaka 565-0871, Japan; 2 Graduate School of Information Science and Technology, Osaka University, Suita-shi, Osaka 565-0871, Japan; 3 Advanced Research Centers, Keio University, Minato-ku, Tokyo, 108-8345, Japan; 4 Graduate School of Integrated Arts and Sciences, Higashihiroshima-shi, Hiroshima, 739-8521, Japan

## Abstract

People sometimes experience a strong emotional response to artworks. Previous studies have demonstrated that the peak emotional experience of chills (goose bumps or shivers) when listening to music involves psychophysiological arousal and a rewarding effect. However, many aspects of peak emotion are still not understood. The current research takes a new perspective of peak emotional response of tears (weeping, lump in the throat). A psychophysiological experiment showed that self-reported chills increased electrodermal activity and subjective arousal whereas tears produced slow respiration during heartbeat acceleration, although both chills and tears induced pleasure and deep breathing. A song that induced chills was perceived as being both happy and sad whereas a song that induced tears was perceived as sad. A tear-eliciting song was perceived as calmer than a chill-eliciting song. These results show that tears involve pleasure from sadness and that they are psychophysiologically calming; thus, psychophysiological responses permit the distinction between chills and tears. Because tears may have a cathartic effect, the functional significance of chills and tears seems to be different. We believe that the distinction of two types of peak emotions is theoretically relevant and further study of tears would contribute to more understanding of human peak emotional response.

Emotion is an essential factor of human life. People experience emotion daily during interpersonal communication, a good or bad workday, and leisure activities. People also experience emotion in response to art, film, and music. Such emotion sometimes becomes intense, giving a peak emotional experience^1^. Some people describe such an experience as ‘being moved’^2^ or ‘kandoh’^3^. People seek art, regardless of culture and time, and many people expect such a peak emotional experience when appreciating art. However, research studies into emotion have mainly examined happiness, sadness, fear, valence, and arousal^4^,^5^. Therefore, many aspects of peak emotion are still not understood although the examination of peak emotion is important to further understanding the human emotional experience. The current study takes a new perspective of peak emotional response to music, especially songs.

Chills are one form of peak emotional responses that have been investigated mainly in the domain of music and emotion (for a review, see refs 6 and 7). The chills refer to a set of bodily sensations, such as shivers or goose bumps. Chills occur not only in response to cold air or illness but also to strong emotional experiences^8^. Goldstein^9^, who was the first to study the phenomenon, asked participants about the psychological elicitors of chills. Goldstein’s survey showed that the category of elicitors with the highest frequency was musical passages although scenes in films and great beauty in nature are other typical elicitors. Because such chills are a clear, discrete event and have the advantage of being elicited by music in emotion research, previous studies have examined the psychophysiological responses to music chills by measuring autonomic nervous system activity. To date, empirical studies have repeatedly shown that music chills are accompanied by increasing electrodermal activity (EDA) due to activation of the sympathetic nervous system (SNS^10^,^11^,^12^,^13^,^14^). Further, a recent study suggested that chills are associated with enlarged pupil diameter, and there exists a positive relationship between chills and SNS activity^15^. Brain-imaging studies have also suggested that chills activate reward-related brain regions, such as the ventral striatum, orbitofrontal cortex, and ventromedial prefrontal cortex^16^,^17^. Furthermore, music chills are accompanied by rewarding dopamine release in the caudate nucleus and nucleus accumbens in the striatum^18^. Therefore, the experience of chills seems to produce physiological arousal and reward for the listener.

However, people may experience another type of strong emotional response: music-elicited tears. Goldstein’s survey^9^ already suggested the existence of music-induced tears. The participants reported that chills were accompanied by the feeling of a lump in the throat or incipient weeping. In this paper, we refer to such an emotional response as ‘tears’. Although Goldstein^9^ suggested that chills and tears are similar phenomena, Sloboda^18^ thought that tears were different from chills. Sloboda^19^ found that different musical features are associated with either tears or chills. By using a multivariate statistical method, Silvia and Nusbaum^20^ showed that peak emotional responses to art have multiple dimensions. Furthermore, a study of ‘being moved’ suggested that tears and chills involved different factors^21^. The study of tears (or crying) is even an independent emotion research domain (for a review, see ref. 22). Considering these studies, it appears that the psychological construct of music-elicited tears is independent of music chills although also somehow similar to it.

The psychophysiological responses of music-elicited tears may be different from those of music chills. In previous studies of emotional tears that examined physiological responses, researchers used film to evoke the tears response^23^,^24^,^25^,^26^. These studies suggested that tears evoked by film produced increasing heart rate (HR) and EDA. Such physiological changes indicate physiological arousal through the activation of the SNS. However, these studies also demonstrated that tears decreased respiration rate (RR^23^,^24^,^26^). Such a physiological pattern indicates physiological calming, which may result from the activation of the parasympathetic nervous system (PNS). As such, music-elicited tears may induce not only physiological arousal but also physiological calming^27^ whereas previous studies have confirmed that music chills activate only the SNS^6^.

Furthermore, the emotional response of music-induced tears may be distinct from that of film-induced tears. Vingerhoets and Bylsma^21^ proposed that tears might have a cathartic effect. Their review reported that tears provide a release of tension and feeling of relief: a pattern captured by the term ‘catharsis’ in questionnaire survey studies. On the other hand, laboratory studies using film indicated that tears were accompanied by increased distress and did not produce any immediate mood improvement although the positive effect of tears is hypothesised^28^. So far, tears studies have shown inconsistent results in terms of emotional responses. However, it is possible that music-elicited tears resolve this inconsistency. Because people often experience sadness when they cry^29^, experimental studies have used sad film as stimuli. As the main character has great ill luck in such films, the viewers find it difficult to experience relief or pleasure although they do have an interest in it (cf. ref. 30). Yet, many studies have shown that sad music can induce a pleasurable feeling^31^,^32^,^33^. Therefore, tears induced by sad music may be different from tears induced by a sad film. Moreover, music is one of the major elicitors of tears^29^. When sad music evokes tears in experimental settings, such tears may be accompanied by pleasure and physiological calming (i.e. cathartic effect).

The present study investigated the psychophysiological responses of two types of peak emotions: chills and tears. We used music as the stimuli because the chills response has been confirmed in music and emotion studies. Further, before the experiment, most participants reported that peak emotional responses were induced by songs (see Methods); therefore, we used only songs as stimuli in the current experiment. The chills and tears responses were measured by self-report sensations during song listening. We conducted an experiment measuring subjective emotions and autonomic nervous system activity. The hypothesis was that tears would be different from chills in terms of both psychological and physiological responses. With respect to psychophysiological responses, we predicted that chills would induce subjective pleasure, subjective arousal, and physiological arousal whereas tears would induce subjective pleasure, relaxation, and physiological calming. In addition, we asked participants to rate song expression in terms of happiness, sadness, calm, and fear in order to understand the emotional property of chills-inducing songs and tear-inducing songs.

## Results

### Manipulation check

The number, intensity, and duration of peak emotional responses for each condition and each group are shown in Table 1. The participants in each group reported the number and intensity of chills or tears responses to be greater for self-selected songs than for experimenter-selected songs. These results confirmed that the participants experienced peak emotional responses for self-selected songs but not for experimenter-selected songs. The intensity and number of chills for the chills group did not differ significantly from the intensity and number of tears for the tears group (intensity: *t*(131) = 0.81, *ns*; number: *t*(131) = −1.10, *ns*), but the duration of chills for the chills group was shorter than the duration of tears for the tears group (*t*(131) = −3.85, *p* < 0.001). The pre-experiment baseline of the physiological activities was also tested for between-group effects. As summarised in Table 2, the physiological activities during the pre-experiment periods did not differ.

### Overall and around peak onset psychophysiological responses

**Figure 1:**
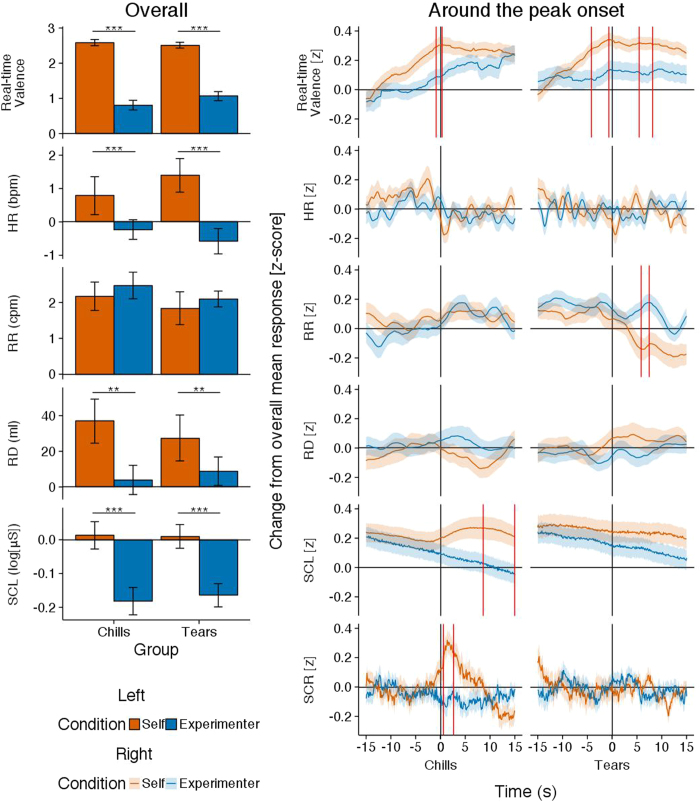
Mean real-time valence ratings and physiological measures for self-selected songs and experimenter-selected songs for the chills and tears peak emotion groups. The left-hand figure shows the overall mean responses. Error bars represent standard error of the mean. The right-hand figure shows the 30 s around the peak onset responses. Lines indicate the mean with the corresponding standard error of the mean. Regions surrounded by the vertical red lines indicate significant differences zones (*p* < 0.05). Note. Self = self-selected song, Experimenter = experimenter-selected song, HR = heart rate, RR = respiration rate, RD = respiration depth, SCL = skin conductance level, SCR = skin conductance response.

Figure 1 (left) shows the overall responses of real-time valence, HR, RR, respiration depth (RD), and skin conductance level (SCL) in response to self-selected songs and experimenter-selected songs separately for the chills and tears groups. Table 3 (left) summarises the results of the two-way analysis of variance (ANOVA) for each of the above variables. Table 3 (right) summarises the tests of significant deviation from the neutral score of the valence rating and baseline of physiological reactivity. The two-way ANOVAs revealed a main effect of song condition on real-time valence, HR, RD, and SCL. All of the main effects showed that self-selected songs were higher for the above variables than experimenter-selected songs, irrelevant of the group. A one-sample *t*-test revealed that real-time valence was higher than neutral for both song conditions in both groups. The HR increased from baseline only for self-selected songs in the tears group. The RD increased from baseline for self-selected songs in both groups, whereas SCL decreased from baseline for experimenter-selected songs in both groups. Moreover, RR increased from baseline for both song conditions in both groups.

Figure 1 (right) shows the 30 s around the peak onset real-time valence, HR, RR, RD, SCL, and skin conductance response (SCR) waveforms in response to self-selected songs and experimenter-selected songs separately for the chills and tears groups. The functional ANOVA (fANOVA; that is, time series analysis^34^) was conducted on the continuous functions for the two conditions. Regions of the vertical red lines show significant differences across the self-selected songs and experimenter-selected songs. The fANOVA were the formal equivalent of planned Tukey LSD post hoc comparisons for each 100 ms time window, with a significance threshold of *p* < 0.05. In the chills group (DF = 1, 132; *p* < 0.05), the real-time valence rating for self-selected songs was significantly higher than for experimenter-selected songs between −0.9 and 0.4 s. The SCL for self-selected songs was significantly higher than for experimenter-selected songs between 8.6 and 15 s. The SCR for self-selected songs was significantly higher than for experimenter-selected songs between 0.6 and 2.7 s. In the tears group (DF = 1, 130; *p* < 0.05), the real-time valence rating for self-selected songs was significantly higher than for experimenter-selected songs between −4.2 and −0.6 s, and between 5.4 and 8.1 s. The RR for self-selected songs was significantly lower than for experimenter-selected songs between 5.9 and 7.5 s. The HR and RD responses were not significantly different in both groups. In addition, the real-time valence for both the chills and tears groups, and SCL and SCR for the chills group, were higher than the overall mean (i.e. 0) during the significant difference period. Whereas the RR for the tears group was lower than the overall mean during the significant difference period, it was higher than the overall mean before this period.

### Emotional rating after listening

**Figure 2:**
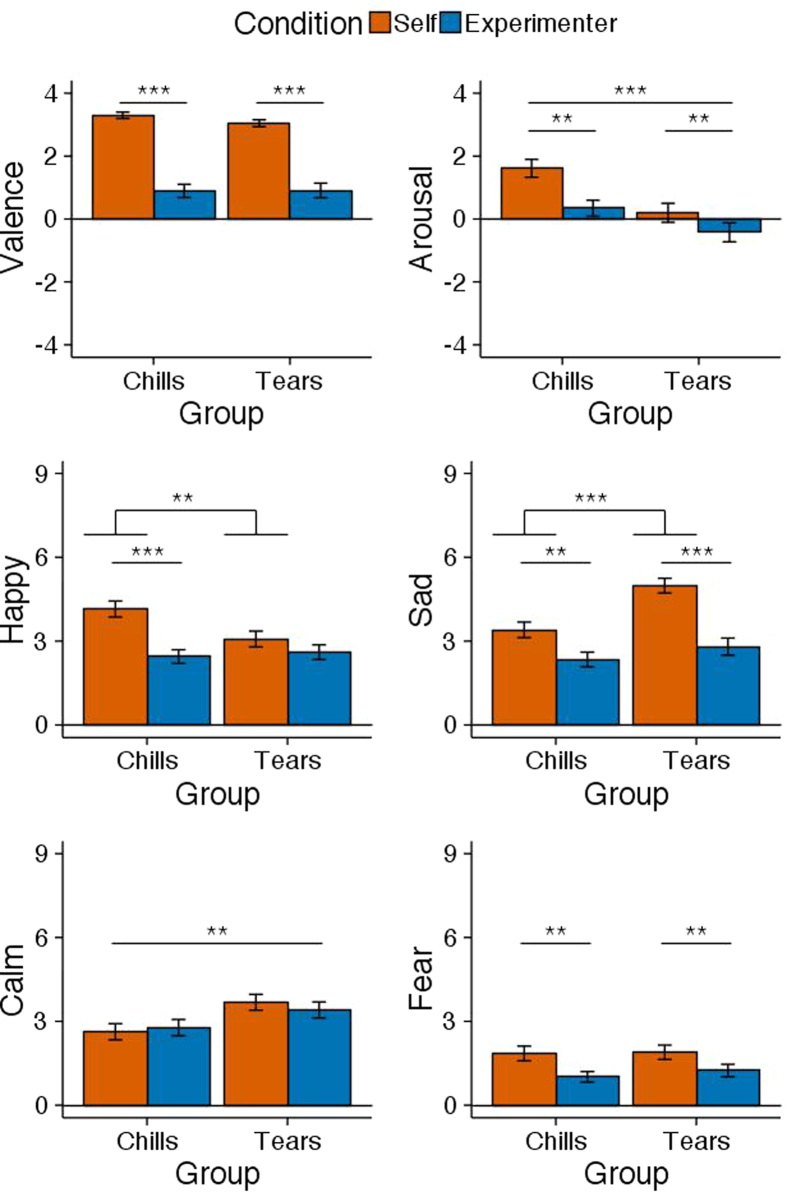
Mean emotional ratings after listening to self-selected songs and experimenter-selected songs for each peak emotion group (chills and tears). Error bars represent standard error of the mean. Note. Self = self-selected song, Experimenter = experimenter-selected song.

Figure 2 shows the emotional ratings of valence, arousal, happiness, sadness, calm, and fear in response to self-selected songs and experimenter-selected songs separately for the chills and tears groups. The results of the two-way ANOVAs and one-sample *t*-test are summarised in Table 4. As shown in Table 4 (left), the two-way ANOVAs revealed a main effect of song condition on valence, arousal, happiness, sadness, and fear. All of the effects showed that the ratings were higher for self-selected songs than for experimenter-selected songs in both groups. The main effect of group emerged on arousal, sadness, and calm. This effect indicated that the chills group was more aroused than the tears group regardless of the song condition whereas the tears group showed more sadness and calm than the chills group regardless of song condition. As the group × song interaction was significant for happiness and sadness, follow-up tests were conducted. For the happiness rating, a simple main effect indicated that self-selected songs were rated higher than experimenter-selected songs in the chills group (*F*(1, 262) = 20.11, *p* < 0.001). The chills group rated higher for happiness than the tears group for the self-selected songs (*F*(1, 262) = 7.95, *p* < 0.01). For the sadness rating, the simple main effect indicated that self-selected songs were rated higher than the experimenter-selected songs in both the chills group (*F*(1, 262) = 7.33, *p* < 0.01) and tears group (*F*(1, 262) = 30.61, *p* < 0.001). The tears group rated higher for sadness than the chills group for the self-selected songs (*F*(1, 262) = 16.21, *p* < 0.001). Furthermore, as shown in Table 4 (right), a one-sample *t*-test showed that valence was higher than neutral for both song conditions in both groups. Arousal was higher than neutral only for self-selected songs in the chills group. Table 5 shows a correlation matrix of subjective emotional reports as a function of group. Intensity, number, and duration of chills were positively correlated with valence, happy, and sad, whereas intensity, number, and duration of tears were positively correlated with valence and sad.

## Discussion

The present study examined peak emotional responses to music, especially songs. So far, research studies of peak emotion have mainly focused on the chills response^6^,^35^,^36^ although Goldstein^9^ already suggested the existence of the tears response. Our main hypothesis was that tears would be psychophysiologically different from chills. As detailed, we predicted that chills would induce subjective pleasure and psychophysiological arousal whereas tears would induce subjective pleasure, relaxation, and physiological calming.

The experimental results showed that the time series responses indicated that chills and tears elicited discriminable physiological responses, although the overall physiological responses were similar for chills and tears. The overall physiological response showed that self-selected music evoked higher HR, RD, and SCL than experimenter-selected music. The overall physiological response was also characterized by significant increases from baseline of RD for chill-inducing songs and increases from baseline of RD and HR for tear-inducing songs. Both group’s response pattern reflects physiological arousal, therefore, it is difficult to find differences between chills and tears. However, around the peak onset, chills were accompanied by physiological arousal due to increases of SCL and SCR similar to previous results of Western and Eastern cross-cultural studies^12^,^13^,^18^, whereas tears were accompanied by psychophysiological calming because the EDA measure did not increase, there was decreased RR^27^,^37^. Because overall HR and RD increased when listening to tear-inducing songs, the slow respiration indicates that there is physiological calming during physiological arousal as found with the psychophysiological responses of relief (for a review, see ref. 38). No previous study has reported such a response with chills. As we used the same method for the chills and tears groups, the differences of the two physiological responses are not caused by a difference in the experimental method.

One might suggest that the physiological effects of the onset of chills and tears derived from emotional characteristics of the music, such as whether it was ‘happy’ or ‘sad’. However, we propose that the influence of musical characteristics is weak or non-existent. First, we cancelled the influence of acoustic features (see Methods). Therefore, the differences in physiological responses between self-selected music and experimenter-selected music did not reflect different acoustic features that elicited emotion in the listener. Second, although chills were related to happy and sad music-related emotions in this study, previous studies showed that music chills are accompanied by increasing SCL and SCR, even when using sad music as stimuli^6^,^14^. That is, the psychophysiological effect of chills does not change contingent on whether music is happy or sad. Third, our results showed that tears-related variables were only correlated with sad (or calm) ratings, and acoustic features of tear-inducing music were more sedative than chills-inducing music (see Table S2). As such, happy music did not generally evoke tears. These observations indicate that physiological arousal and calming are not due to emotion-related musical characteristics, but rather due to each of the two peak emotions, namely chills and tears. However, as previous questionnaire studies suggested, chills and tears could be co-activated^9^,^19^. This suggests that some chills and tears responses included the other response, especially for sad music. Since we did not measure the tears response in the chills group or the chills response in the tears group, both effects could be confounded. Future studies should examine the independence of chills and tears by measuring both chills and tears responses in each group. Such an examination would confirm the psychophysiology of chills and tears responses.

Moreover, the subjective responses also reflected the difference of the two peak emotions. Although the real-time valence rating (and their mean) indicated similar pleasurable response for chills and tears, the overall emotional rating suggested that chills involved subjective pleasure and arousal from both happy and sad songs, whereas tears involved pleasure from sad songs. Tear-inducing songs were calmer than chill-inducing songs, and the tears sensation showed longer duration than the chills sensation. These results almost corresponded with our hypothesis; therefore, the current results indicate that music-elicited tears are another peak emotional response distinct from music chills. Sloboda^19^ thought that chills are a different psychological response from tears whereas some studies have proposed that chills and tears are the same peak emotional response^9^,^39^. Therefore, it was unknown whether the two types of responses are the same or different. Our study is the first to show that chills and tears are different phenomena from their psychophysiological responses. In addition, psychoacoustic features of chill-inducing music were also different from those of tear-inducing music (Table S2).

When the current findings are compared to previous tears studies, they seem to demonstrate the tears’ function of catharsis. Reviews of previous tears studies^22^,^27^ showed that an arousing distress effect was found in experimental settings whereas questionnaire surveys supported the catharsis effect. Our experiment resolved this inconsistency because the tears response was accompanied by physiological calming during physiological arousal and an increased feeling of pleasure from an intensely sad song. Although the physiological responses are somehow similar to those of arousing distress tears^23^,^24^, the current response captures the characteristics of catharsis that give a release of tension and feeling of relief. Contrary to our hypothesis, tears did not induce subjective relaxation (deactivation) probably because tears evoked a temporary physiological calming effect during the general physiological arousal. However, tears from sad songs did induce a clear pleasurable feeling for both the overall and around peak onset responses. As such, it is safe to say that the current study shows the cathartic effect of tears in an experimental setting. Previous experimental studies of tears that used intolerable tragedy films (such as films depicting death and murder) as stimuli did not support the cathartic effect^23^,^25^. As the present study used participants’ favourite sad song, it is possible that a favourite sad film could also easily evoke cathartic tears and that the sad song could evoke cathartic tears more easily than a sad film. The former possibility should be examined in a future study. Because a sad film often wins a big market and many people like to see it, a favourite sad film could evoke cathartic tears rather than distress tears. We think that the latter is also a possibility because other people expressing sadness visually appear in a sad film whereas other sad people do not directly appear in a sad song. Therefore, it may be more difficult for a sad film to induce a pleasurable experience than a sad song. Saarikallio and Erkkilä^40^ and Lamont^41^ reported cases in which listeners could identify with the sad character of the sad song and felt as if the singer knew their own sad experiences, making them feel understood and bringing pleasure. We speculate that such identification could be possible because music involves imagining things for simply a sequence of tones unfolding over time resulting in a vague border between the listener and character in the song. This property of music could be related to a role of tears that involves individuals re-experiencing their past sadness and helping them to overcome it^28^. These psychological processes may be important to the experience of cathartic tears.

The two types of peak emotional responses are also different in terms of functional significance. Past studies showed that chills produce psychophysiological arousal through SNS activation and a rewarding effect through the activation of reward-related brain regions, such as the medial orbitofrontal cortex and nucleus accumbens^16^,^18^. On the other hand, the current results indicated that tears could produce physiological calming and a cathartic effect. It is possible that tears responses are also related to PNS activation^42^. Although the brain responses of the cathartic tears are still unknown^29^, the reward function and catharsis function may have different benefits for humans. Humans retain the two types of peak emotional responses probably because of two different types of functional significance. Art, including music, is not necessary for survival and does not have biological advantages, as food and sex do, but people have sought art through all of the ages and over the whole world. The function of chills and tears may be one reason why people need music and other art.

Both types of peak emotional response could be characterised by mixed emotion^43^ rather than basic emotions and two emotional dimensions^4^,^5^. When people experience mixed emotion, they simultaneously feel a positive and negative emotional state. A meta-analysis confirmed that mixed emotion studies have demonstrated some types of simultaneous positive and negative activation (e.g. happy and sad, amusement and disgust) for personal experiences, films, and music^44^. Mixed emotions can occur even during the same section of films and music^45^,^46^. Already, some chills studies have suggested a positive relationship between chills and mixed emotions (happy and sad)^6^,^35^,^47^. The current results found that the mixed emotion of chills was simultaneous pleasure, happiness, and sadness. This finding means that chills provide mainly a positive experience but the sadness factor is necessary even though a favourite song is the elicitor. Given that music chills activate reward-related brain regions^16^,^18^, such an emotional property could make chills a unique experience and separate chills from other mixed emotional experiences. Furthermore, as the mixed emotion of tears was simultaneous pleasure and sadness, it was different from the mixed emotion of chills. The tears response contributes to the understanding of the pleasure of sad music. As people generally feel displeasure for sad things, this is a unique mixed emotional response with regard to music^48^. Although previous studies showed that sad music induced relatively weak pleasure^31^,^32^, the current tears’ results showed that sad songs induced strong pleasure. It is difficult to account for why people feel sad music as pleasurable^49^,^50^,^51^; however, the current results suggested that the benefit of cathartic tears might have a key role in the pleasure generated by sad music. Therefore, the two types of peak emotional responses may uniquely support knowledge of mixed emotion.

Note that co-activation of arousal and calm is difficult to understand from the perspective of mixed emotion. Chills-group participants showed calm and activation for self-selected music, and tears-group participants showed calm and neither activation nor deactivation for self-selected music. These relationships between arousal and calm seem contradictory. A review of mixed emotions suggested that people experience two oppositely valenced emotions^44^; however, no evidence exists for two emotions with opposing levels of arousal. Because the chills group produced higher arousal ratings than the tears group, while the tears group showed higher calm ratings than the chills group, and arousal was negatively associated with calm in both groups (Table 5), participants properly recognized the inverse relationship between arousal and calm. It is possible that participants may report one song part as calm and another song part as arousing, because a song can have several parts that elicit different emotions^52^. Future studies should examine such overall rating contradictions by using real-time arousal ratings. Arousal and calm may emerge in different song sections.

However, the current findings are limited in some respects. First, as we used a between-subjects design for measuring the chills and tears responses, the individual differences of participants may influence our results. It is possible that the participants in the chills group experience physiological arousal easily whereas the tears group may be sensitive to physiological calming. To show the different psychophysiological effects of chills and tears more generally, we should conduct the experiment using a within-subjects design and collect chills and tears responses from the same participant. Second, we used only songs in the current experiment. As such, it is highly likely that both acoustic features and lyrics influenced emotional responses. Previous studies have confirmed that instrumental music can evoke a chills response^11^,^14^,^18^, and Sloboda^19^ suggested that instrumental music can evoke a tears response. However, we do not know whether a tears response can be evoked in experimental settings by instrumental music. Further, we do not know whether psychophysiological response differences between chills and tears would emerge via instrumental music. It remains an open question as to whether the results can be generalized to instrumental music. Third, the song stimuli were familiar to the participants, which is why the experimental stimuli have familiarity and memory effects. Such cognitive factors could influence the psychophysiological responses. We revealed *what* responses were induced by chills and tears, but novel musical stimuli will be needed to show *why* chills and tears were evoked by the music. Fourth, musical emotion may have complex characteristics and the Geneva Emotional Music Scales (GEMS) are needed to measure the full range of emotions^53^. Although it may be premature to use GEMS except for with classical music^54^, the nostalgia, transcendence, and wonder emotions included in the GEMS might show further emotional differences between chills and tears. Finally, we measured chills and tears responses using subjective reports. Subjective reports are not always correct and it is difficult to guarantee the same measure from all participants. Benedek *et al*.^55^ have obtained an image of the skin on the arm by video camera and observed the piloerection. No study has developed an objective method of measuring tear responses. Although the moisture of tears could be measured using litmus paper, online tear measurement is difficult, and innovative method remain to be developed. Future research should use such an objective index for both chills and tears to elucidate the biological differences of the two responses.

The aim of the study was to reveal the existence of two types of peak emotional responses to music. We targeted responses to songs and showed that chills and tears can be attributed to different psychological factors. Furthermore, chills induced psychophysiological arousal (increased EDA and deep breathing) and pleasure from both happy and sad songs, whereas tears induced physiological calming during physiological arousal (slow, deep breathing and accelerated HR) and pleasure from sad and calm songs. Until now, peak emotion studies have been conducted mainly of chills^3^,^5^,^6^ and experimental studies have found that tears responses are treated as a distressing negative emotion^29^. The current study introduces the new perspective of the peak emotional response of pleasurable cathartic tears. Further study of both chills and tears will contribute to better understanding of human emotion.

## Methods

### Participant screening

A total of 154 participants completed a questionnaire survey to assess the frequency of chills and tears for everyday listening. All participants were Japanese-speaking students and the complete study was conducted in Japanese. They were recruited from some regularly scheduled psychology classes in a university. Participants answered four questions as follows: ‘While listening to music, how frequently do you (1) get goose bumps, (2) feel shivers down your spine, (3) feel like weeping, and (4) get a lump in your throat’? The four items were rated on a Likert response scale that ranged from 0 (*not at all*) to 10 (*nearly always*). The scores of questions (1) and (2) were averaged as a chills score (Cronbach’s α = 0.74), while the scores of questions (3) and (4) were averaged as a tears score (Cronbach’s α = 0.75). Furthermore, participants provided the title of a musical piece that evoked a strong emotional response. We separately gathered chills- and tears-group participants because previous studies did not measure music-elicited tears in experimental settings. To enable participants to be fully engaged in the tear-evoking task and to increase the likelihood of obtaining tears responses, we conducted tears and chills experiments in several groups, using a between-participants design. In addition, both experiments required 90 minutes to complete; participants also did not take part in both conditions to avoid fatigue. Eleven participants were not asked to take part in the experiment due to their report that they have never experienced chills or tears during music listening. Most participants (83.2%) reported a Japanese pop/rock song as a chills or tears elicitor, while 24 participants reported classical or instrumental music (12.6%) or a UK/US song (4.2%). In order to maintain music characteristics, we only included the participants who reported that a Japanese pop/rock song evoked a strong emotional response. Moreover, 29 participants were not suitable to participate in the experiment because they could not select three pieces of music that had previously elicited a chills or tears response, and six participants were excluded due to difficulty with matching the control song (see the Stimuli section). An additional 18 participants were originally selected to be part of the chills group (10 participants) or tears group (eight participants), but were excluded from the analysis because they did not report chills or tears during the experiment. The remaining 66 participants were analysed.

### Participants

Thirty-two undergraduates participated in the chills group experiment (14 males and 18 females; mean age 18.84 years, *SD* = 0.77) and thirty-four undergraduates participated in the tears group experiment (13 males and 21 females; mean age 18.79 years, *SD* = 1.07). For each group, we selected participants who reported at least one experience of chills or tears for music. We assigned the participants who showed a relatively high score for chill frequency to the chills group, and assigned the participants who showed a relatively high score for tear frequency to the tears group. In the chills group, the mean score of chill frequency was 4.33 (*SD* = 2.27) and tear frequency was 2.77 (*SD* = 2.02). In the tears group, the mean score of chill frequency was 2.66 (*SD* = 1.99) and tear frequency was 4.65 (*SD* = 1.91). Musical experience between the chills group (*M* = 7.91, *SD* = 7.05) and tears group (*M* = 6.50, *SD* = 5.04) was not significantly different (*t*(64) = 0.94, *ns*). We calculated the years of musical experience by summing up years of experience across all instruments and vocals (e.g. 3 years of experience with the piano and 2 years with the guitar = 5 years of total musical experience). Participants were required to be in good general health to participate in the study. We assessed that participants did not have a history of neurological, psychiatric, or cardiovascular disorders, or chronic medical conditions. All participants were instructed to abstain from coffee or alcoholic beverages the night before the physiological response measurement. Participants were compensated for their participation (about US$8 and course credit).

### Stimuli

Participants were instructed to select their three favourite songs before the experiment. Each of the 32 or 34 participants selected three songs that had previously elicited a chills or tears response. These 96 songs in the chills group and 102 songs in the tears group were kept separate. Then, 198 songs were used as stimuli in the present experiment (all songs are listed in Table S1, and the musical features extracted by music information retrieval method in Table S2). After collecting all of the song information before the experiment, the experimenter prepared digitalised files (WAV file format) from the original CD recordings. To maintain ecological validity, the song stimuli were the full-length version of the songs including the lyrics. The mean song duration was 295.7 s in the chills group and 304.4 s in the tears group. Because one’s favourite song shows a high probability to evoke a strong emotional response in the laboratory^18^,^56^, we used such songs as the experimental stimuli.

Each participant listened to six songs, including the test and control song. The test songs were three self-selected songs, whereas the control song was selected for each individual by applying methods in which one participant’s peak emotional song was used as another participant’s control song^14^,^18^,^57^. The experimenter matched the control song for each participant within the chills group or tears group. In this matching procedure, we avoided a given participant’s six favourite artists (the three artists who performed the self-selected song and their three favourite artists, which was ascertained prior to the experiment) in order to suppress a potential peak emotional response evoked by the control song. For example, if song set A (three songs) evoked peak emotions in participant 1, and song set B (three songs) evoked peak emotions in participant 2, then song set B served as the psychoacoustic control for participant 1, and song set A served as the psychoacoustic control for participant 2. In the experiment, each of the 96 songs in the chills group and 102 songs in the tears group was used once as a self-selected song and again as an experimenter-selected song. By using this matching procedure, we collected 33 pairs of data from 66 participants (16 pairs of data from 32 participants in the chills group and 17 pairs of data from 34 participants in the tears group). The advantages of this matching method were that it allowed us to analyse data by comparing the same sets of stimuli and to exclude the possibility that found effects are solely due to the acoustic changes of the song (e.g. sudden increase in tempo or elevations in pitch).

### Recordings of physiological signals

Three physiological signals were recorded: an electrocardiogram (ECG), respiration, and EDA. The ECG was recorded using three disposable Ag/AgCl 38 mm diameter spot electrodes positioned in a three-lead chest configuration (right collarbone, lowest left rib, and lowest right rib). The RR and RD were measured with a piezoelectric transducer belt placed around the chest. The EDA was measured on the palmar surface of the middle phalanges of the first and second fingers of the left hand (DC, time constant 4 s). A constant-voltage device maintained 0.5 V between 19 mm Ag/AgCl electrodes filled with sodium chloride gel. The ECG and respiration were acquired with a Polygraph 360 (NEC Medical Systems, Japan) and EDA was obtained with a DA3-b sensor (Vega Systems, Japan). The recordings of the three physiological signals were sampled at 1 kHz using a Vital Recorder Monitoring System (Kissei Comtec, Japan).

### Procedure

The listening experiment was conducted inside a sound-attenuated chamber and stimuli were presented through two loudspeakers (DS-200ZA; Diatone, Japan) positioned in front of the participants. The loudspeakers were connected to a computer outside of the chamber via an amplifier (PM-7SA; Marantz, Japan). Experimental tasks were controlled with the customised Visual C++ 2008 programme (Microsoft, USA). The synchronisation of experimental tasks and physiological measures was also controlled with the programme. The temperature in the room was kept at 24 ± 1 °C. The experiment was conducted with one participant at a time. After obtaining informed consent, the participant was seated in front of a computer screen and attached to the physiological sensors, such as the electrodes and the respiration belt. Then, they were asked to sit still, not to speak, and to relax with their eyes open during a 7-min laboratory adaptation period. Following this, a 3-min pre-experiment baseline of physiological activities was measured. All of the participants took part in a training session to become familiar with the experiment before the main trial.

In the main trial, each participant listened to six songs in a pseudo-randomised order across the test pieces and control pieces. In order to avoid three self-selected or control pieces presented continuously, we used a pseudo-randomised method with at least one self-selected (control) song presented between the three pieces of control (self-selected) song. The song stimuli were presented at each participant’s comfortable listening level because peak emotional responses are readily elicited by this listening state^14^. In order to keep track of their peak emotional responses during song listening, participants were asked to complete button-press measures. In the chills group, this involved pressing the left button of a computer mouse whenever they felt chills (instructed as ‘goose bumps’ or ‘shivers down the spine’). In the tears group, this involved pressing the left button of a mouse whenever they felt tears (instructed as ‘weeping’ or ‘a lump in the throat’). Importantly, the only difference in procedure between the chills and tears groups was the psychological index of the mouse-button click, which enabled the comparison of the physiological effect of chills and tears without experimental artefacts. Participants were also asked to rate their subjective valence level in real time via movements of the mouse exhibited on the computer display at 480 pixel scale. Moving the mouse to the right indicated heightened inner pleasure, whereas moving the mouse to the left indicated heightened inner displeasure. If participants did not feel pleasure or displeasure, they moved the mouse to the centre. The mouse-button signals and valence ratings were recorded with a 10-Hz sampling rate. After listening to each song, participants rated how intensely they felt chills or tears in the overall song. Both responses were given on a 9-point Likert-type scale ranging from 0 (*not at all*) to 9 (*very strong*). Participants also rated the felt emotional responses to each song in terms of valence (−4 = displeasure, 0 = neutral, 4 = pleasure) and arousal (−4 = deactivation, 0 = neutral, 4 = activation). Furthermore, they rated the song in terms of happiness, sadness, calm, and fear on a scale ranging from 0 (*not at all*) to 9 (*very strong*). We selected these four emotions because they encompass the four quadrants of a two dimensional emotional space (happy: high-arousal, high-valence; calm: low-arousal, high-valence; fear: high-arousal, low-valence; sad: low-arousal, low-valence). Previous music and emotion studies showed that music can simultaneously evoke happy and sad emotions (i.e. mixed emotions) but not pleasure and displeasure^44^,^45^; therefore, the happy-sad dimension may not be the equivalent of the pleasure-displeasure dimension in musical emotions^58^,^59^. We measured happy, sad, calm and fear perceived emotions in order to capture the quality of musical emotion more precisely than only two dimensional models, and we expected to receive some mixed emotional responses (i.e. happy-sad, happy-fear, and calm-sad). After this, the participant was asked to relax for 1 min as a rest period. We set the latter 30 s of this period as the physiological baseline for the subsequent song stimuli. The experimental procedure was approved by the Faculty of Integrated Arts and Sciences Ethics Committee of Hiroshima University. We confirmed that all experimental methods were performed in accordance with relevant guidelines and regulations.

### Quantification of the time series data

For the emotional evaluation, the onset of peak emotional response was defined as the start time when participants pressed the mouse button, which was the chills response in the chills group and the tears response in the tears group. The number and duration of button presses were counted for each song piece, and these were used as measures of the number and duration of peak emotional experiences. Because we considered button presses that occurred less than 1 s after a previous press to not be peak emotional responses, we removed these from the analysis. The first 15 s of peak emotional responses were also not included in the analysis to avoid a physiological initial orientation response^60^,^61^. Furthermore, the values of the real-time valence ratings were divided by 60 in order to set the same range of valence ratings after listening.

For the physiological signals, HR (beats per minute) was determined by a programme that detects R spikes in the ECG (Peakdet, Version 3.4.305^62^; for use in MATLAB, MathWorks, USA) and calculates interbeat intervals (IBIs). Beat-to-beat values were edited to exclude outliers due to artefacts or ectopic myocardial activity. Outlier IBI values were identified by flagging intervals that were larger than 1,500 ms or 150% of the mean value of the preceding 10 intervals, or smaller than 500 ms or 50% of the mean value of the preceding 10 intervals (an outlier of approximately 0.1%). Linear interpolated R spikes were inserted when the IBI was too long, whereas R spikes were deleted when the IBI was too short. Then, to obtain the time series data, the cubic spline interpolation of the non-equidistant waveform of the IBI sequence was completed, and IBIs were resampled at 10 Hz in order to synchronise the time series with the subjective responses. A similar computer-assisted procedure for detecting respiration peaks was applied to the respiration signal. The moments of maximal inspiration and minimal expiration were used to determine RR (in cycles per minute) and RD (calculated by the amplitude from base to peak of a single ventilator cycle^63^). The detected peaks were edited for outliers (respiratory cycles <1,000 ms). Both respiratory parameters were interpolated with a cubic-spline function and resampled at 10 Hz, similar to the HR. Furthermore, SCL and SCR from the electrodermal data were downsampled to 10 Hz. SCL reflects tonic sympathetic activity and has a relatively long latency and changes slowly. In contrast, SCR reflects phasic sympathetic activity, emerges within 1–3 s, and changes quickly^64^. They were expressed in microSiemens (μS). To account for the typically skewed distribution of electrodermal response measures, the SCL and SCR values were each transformed into log and log + 1^64^.

### Analysis strategy

We first conducted a manipulation check. To confirm the validity of the experimental condition, the degree of peak emotion evoked for the self-selected song and experimenter-selected song was compared. To test for the same level of elicitation of peak emotion, we compared the chills measure in the chills group and tears measure in the tears group. Furthermore, we examined whether the pre-experiment baseline of physiological activities between groups was the same or not.

For the main analysis, we tested overall responses and the 30 s around the peak onset of responses for the psychophysiological measures. Overall responses scores were derived for each rest and song period. Difference scores were calculated by subtracting the average from the 30 s immediately preceding the rest period from each of the 10 Hz physiological data of the song period. The difference scores were then averaged within each song period. In addition, the overall valence score was calculated as the mean of the real-time valence rating during each song period. For the 30 s around the peak onset of responses, we used the *z*-score derived from each song period. The *z*-scores were calculated of the 10 Hz difference scores of physiological responses and real-time valence ratings. The *z*-score represents the relative change of overall mean response to the stimulus. The positive value of a *z*-score reflects an increase from the mean score of the overall response, whereas the negative value of a *z*-score reflects a decrease from the mean score of the overall response. To show whether the chills and tears effects stand out during song listening, we tested the psychophysiological responses to around the chills or tears period using the *z*-score. The physiological responses and real-time valence ratings for self-selected songs were included in an ‘around peak onset response window’ ranging from 15 s before to 15 s after the chills or tears onset per song stimulus. We set this time window because some previous studies have shown that psychophysiological responses during this time window are sufficient to examine the chills experience^6^,^10^,^14^.

We created matching trials in order to control for the effect of acoustic changes on the psychophysiological responses. That is, the trials during which participants experienced chills or tears for self-selected songs were matched with the trials of the same song that were heard by a different participant as an experimenter-selected song. Importantly, this matching procedure was conducted within the experimental group for chills or tears. The overall responses for both the self-selected song and experimenter-selected song were averaged for the number of times based on the chills- or tears-induced trial for the self-selected song. The 30 s around peak onset windows for both self-selected songs and experimenter-selected songs were first averaged for the number of peak emotion times for each chills- or tears-induced trial for self-selected songs. Subsequently, these data were further averaged in the same way as the overall responses. Therefore, the same number of psychophysiological responses for a song was collected for each self-selected song and experimenter-selected song.

### Statistical analysis

All statistical analyses were performed with the R 3.3.1 software^65^. As a manipulation check, the effect of song condition on peak emotional responses was investigated using a between-subjects *t*-test. The dependent variables were the number, intensity, and duration of chills in the chills group and of tears in the tears group. Next, between-subjects *t*-tests were calculated to test whether the above chills-related measures in the chills group differed from the tears-related measures in the tears group. In addition, we compared the chills group with the tears group for the pre-experiment baseline of physiological activities using between-subjects *t*-tests.

The overall psychophysiological responses were analysed using ANOVA. A 2 (self-selected song vs. experimenter-selected song) × 2 (chills group vs. tears group) between-subjects ANOVA was conducted using the dependent variables of real-time valence and each physiological measure. In addition, we used a one-sample *t*-test to assess whether these parameters deviated from zero separately for each of the four ANOVA levels. Furthermore, in order to analyse the time series psychophysiological data, we used functional data analysis techniques^34^. The functional data analysis techniques were specifically developed for analysing temporal data^66^. In these techniques, mathematical functions are first fitted to the discrete 10 Hz data. Next, a statistical analysis was performed on the continuous function. Fourth-order (cubic) B-splines were fitted to each individual’s raw psychophysiological responses with 30 basis functions to the data samples. The data were smoothed with a constant smoothing parameter (λ = 0.1). This smoothing value was chosen so as not to eliminate the contours of each variable that were important to the analysis. One-way fANOVA were employed with condition (self-selected vs. experimenter-selected) as the between-subjects variable. A fANOVA was conducted on the real-time valence and each physiological response within the chills or tears group comparison. An advantage of this technique compared to traditional ANOVA is that it allows us to discover ‘when’ the psychophysiological responses were significantly different. Functional *p*-value curves were calculated for each response using a functional permutation *F*-test with 1000 random permutations^34^.

In addition, a two-way ANOVA, which was the same for the overall responses, was conducted using the dependent variables of the subjective emotional rating of valence, arousal, and the perceived song expression rating of happiness, sadness, calm, and fear that were obtained after listening. Again, we used a one-sample *t*-test to assess whether valence and arousal deviated from zero separately for the four ANOVA levels. Correlations between all subjective emotional responses were also performed for chills and tears groups.

### Data handling

We treated peak emotional responses for self-selected songs that exceeded 2.5 standard deviations from the mean as outliers because there were too many of these responses and they were not reliable as peak emotional responses. We excluded trials in which the experimenter-selected song induced more chills or tears than the self-selected song because such trials did not fit our analysis criteria. In addition, programme error forced us to exclude two participants in the chills group. Finally, peak emotional responses were collected and analysed for 67 songs in the chills group and 66 songs in the tears group.

## Additional Information

**How to cite this article**: Mori, K. and Iwanaga, M. Two types of peak emotional responses to music: The psychophysiology of chills and tears. *Sci. Rep.* **7**, 46063; doi: 10.1038/srep46063 (2017).

**Publisher's note:** Springer Nature remains neutral with regard to jurisdictional claims in published maps and institutional affiliations.

## Supplementary Material

Supplementary Information

## Figures and Tables

**Table 1:** Descriptive Statistics for the Two Types of Peak Emotional Responses (Chills and Tears) and the Results of the *T*-Tests for the Self-Selected and Experimenter-Selected Song Conditions.

	Peak emotional response					
	Self-selected		Exp.-selected		t-test	
	*M*	*SD*	*M*	*SD*	*t*	*p*
Chill						
Number	6.92	4.04	0.70	1.47	11.85	<0.001^***^
Intensity	5.45	1.27	1.12	1.27	15.39	<0.001^***^
Duration (s)	3.47	4.17	0.21	0.86	6.27	<0.001^***^
Tear						
Number	6.12	3.57	0.79	1.25	11.45	<0.001^***^
Intensity	5.80	1.63	1.97	1.63	11.05	<0.001^***^
Duration (s)	6.60	5.41	0.71	1.75	8.42	<0.001^***^

Note. *df* = 131. Exp. = experiment. The chills measures were obtained from the chills group and the tears measures were obtained from the tears group. All *p-*values of the correlation coefficients are <0.001.

**Table 2:** Descriptive Statistics for the Pre-Experiment Baseline of Physiological Activity and the Results of the *T*-Tests for the Chills and Tears Groups.

Physiology	Group					
	Chills		Tears		t-test	
	*M*	*SD*	*M*	*SD*	*t*	*p*
HR	72.79	10.40	70.19	10.42	1.02	*ns*
RR	15.15	2.96	16.61	3.13	−1.95	*ns*
RD	290.98	92.96	279.69	64.87	0.57	*ns*
SCL	3.49	0.62	3.52	0.76	−0.17	*ns*

Note. *df* = 64. HR = heart rate, RR = respiration rate, RD = respiration depth, SCL = skin conductance level. All p-values of the correlation coefficients are >0.05.

**Table 3:** Results of the Analysis of Variance and *T*-Tests for the Overall Physiological Reactivity of Response of the Tears and Chills Groups and Self-Selected and Experimenter-Selected Song Conditions.

Psychophysiology	Group		Song		Group × Song		Deviation from baseline or neutral			
	*F*	*p*	*F*	*p*	*F*	*p*	*t_chill-self_*	*t_chill-exp_*	*t_tear-self_*	*t_tear-exp_*
Real-time valence	0.66	0.42	207.28	<0.001^***^	2.16	0.14	29.43 ^***^	5.88^***^	30.22^***^	8.25^***^
HR	0.08	0.77	11.06	<0.001^***^	1.14	0.29	1.38	−0.78	2.77^**^	−1.52
RR	0.91	0.34	0.56	0.45	0.00	0.95	5.49^***^	6.66^***^	4.02^***^	9.48^***^
RD	0.05	0.83	5.93	0.002^**^	0.46	0.50	2.99^**^	0.48	2.13^*^	1.11
SCL	0.04	0.85	23.94	<0.001^***^	0.07	0.78	0.32	−4.50^***^	0.28	−4.78^***^

Note. See Figure 1 (left) for descriptive statistics. For the analysis of variance, *df* = 1, 262 and for the *t*-tests *df* = 66 in the chills group, and *df* = 65 in the tears group. *p < 0.05, **p < 0.01, ***p < 0.001. HR = heart rate, RR = respiration rate, RD = respiration depth, SCL = skin conductance response, chill-self = response for self-selected song in the chills group, chill-exp. = response for experimenter-selected song in the chills group, tear-self = response for self-selected song in the tears group, and tear-exp. = response for experimenter-selected song in the tears group.

**Table 4:** Results of the Analysis of Variance and *T*-Tests for the Emotional Evaluation After Listening to Song in the Chills and Tears Groups and Self-Selected and Experimenter-Selected Song Conditions.

Emotional rating	Group		Song		Group × Song		Deviation from neutral			
	*F*	*p*	*F*	*p*	*F*	*p*	*t_chill-self_*	*t_chill-exp_*	*t_tear-self_*	*t_tear-exp_*
Valence	0.48	0.49	173.05	<0.001^***^	0.60	0.44	33.06^***^	4.27^***^	27.44^***^	3.95^***^
Arousal	14.66	<0.001^***^	10.99	0.001^**^	1.29	0.26	5.70^***^	1.37	0.65	−1.41
Happy	2.89	0.09	16.25	<0.001^***^	5.23	0.02*				
Sad	13.50	<0.001^***^	34.04	<0.001^***^	4.08	0.04*				
Calm	8.64	0.004^**^	0.05	0.83	0.54	0.46				
Fear	0.33	0.56	10.08	0.002^**^	0.15	0.69				

Note. See Figure 2 for descriptive statistics. For the analysis of variance *df* = 1, 262 and for the *t*-tests *df* = 66 in the chills group, and *df* = 65 in the tears group. *p < 0.05, **p < 0.01, ***p < 0.001. Chill-self = response for self-selected song in the chills group, chill-exp. = response for experimenter-selected song in the chills group, tear-self = response for self-selected song in the tears group, and tear-exp. = response for experimenter-selected song in the tears group.

**Table 5:** Correlation Matrix of Subjective Emotional Responses for Chills and Tears Groups.

Measure	1	2	3	4	5	6	7	8	9
1. Intensity	—	0.67^***^	0.66^***^	0.68^***^	0.11	0.09	0.60^***^	0.19^*^	0.16
2. Number	0.77^***^	—	0.62^***^	0.45^***^	0.11	0.07	0.44^***^	0.00	0.15
3. Duration	0.58^***^	0.63^***^	—	0.46^***^	0.16	0.16	0.33^***^	0.12	0.07
4. Valence	0.73^***^	0.58^***^	0.39^***^	—	0.13	0.33^***^	0.41^***^	0.35^***^	−0.02
5. Arousal	0.28^***^	0.13	0.08	0.37^***^	—	0.20^*^	−0.14	−0.40^***^	0.13
6. Happy	0.43^***^	0.34^***^	0.26^**^	0.44^***^	0.56^***^	—	−0.25^**^	0.28^***^	0.05
7. Sad	0.25^**^	0.27^***^	0.22^**^	0.05	−0.15	−0.14	—	0.12	0.31^***^
8. Calm	0.06	0.07	0.10	0.00	−0.38^***^	0.06	0.25^**^	−	−0.11
9. Fear	0.18^*^	0.16	0.14	−0.06	0.11	−0.03	0.53^***^	0.10	—

Note. Inter-correlations for the chills group (*n* = 134) are presented below the diagonal whereas those for the tears group (*n* = 132) are presented above the diagonal. *p < 0.05, **p < 0.01, ***p < 0.001.
