# Supplementary material for: Two types of peak emotional responses to music: The psychophysiology of chills and tears
Source: Sci Rep. 2017 Apr 7;7:46063. doi: 10.1038/srep46063 (PMC5384201; doi:10.1038/srep46063)
Supplement: Supplementary Information [file srep46063-s1.pdf]

Supplementary Information

Two types of peak emotional responses to music: The psychophysiology of chills and tears

*Kazuma Mori[1,2,3] and Makoto Iwanaga[4]

1 National Institute of Information and Communications Technology

2 Osaka University

3 Keio University

4 Hiroshima University

*Kazuma Mori, Center for Information and Neural Networks, National Institute of Information and Communications Technology, Japan, 1-4 Yamadaoka, Suita, 565-0871, Osaka, Tel: + 8090983240, Fax: +671748612, Email: kazumamori@nict.go.jp

Song lists

In our experiment, individual participants selected musical stimuli. Table S1 showed song lists used in the study. All songs are sung by Japanese. They are commercially available in Japan. Because our experiment was conducted Jun to August in 2011, recent some year songs are not included.

Table S1. All songs selection for the current experiment.

Artist	Song title	Participant
Mr. Children	君が好き	chill 1
Superfly	Hello, Hello	chill 1
MISIA	逢いたくていま	chill 1
Nana Mizuki	Pray	chill 2
Suara	キミガタメ	chill 2
YUI	How crazy	chill 2
RADWIMPS	愛し	chill 3
Spitz	めざめ	chill 3
サスケ	卒業の日	chill 3
東京事変	透明人間	chill 4
JUDY AND MARY	くじら 12 号	chill 4
BIGMAMA	Paper-craft	chill 4
電気グルーヴ	虹 (Short Cut Mix)	chill 5
電気グルーヴ	安里屋ユンタ	chill 5
電気グルーヴ	Shangri-La	chill 5
aiko	カブトムシ	chill 6
Mao Abe	貴方の恋人になりたいのです	chill 6
ALI PROJECT	薔薇獄乙女	chill 6
BUMP OF CHICKEN	飴玉の唄	chill 7
Mao Abe	いつの日も	chill 7
コブクロ	Fragilemind	chill 7
Megumi Hayashibara	集結の園へ	chill 8
B'Z	It's show time	chill 8
B'Z	さよなら傷だらけの日々よ	chill 8

PornoGraffitti	Rainbow	chill 9
plenty	枠	chill 9
Anri Kumaki	花言葉	chill 9
INFINITY 16	いつまでもメリークリスマス	chill 10
EXILE	I wish for you	chill 10
flumpool	君に届け	chill 10
ジン	Fuga	chill 11
Kazushi Miyazawa & Jo Hisaishi	旅立ちの時 ～Asian Dream Song～	chill 11
BUMP OF CHICKEN	車輪の唄	chill 11
WEAVER	君と僕のテーマソング	chill 12
WEAVER	心の中まで	chill 12
WEAVER	白朝夢	chill 12
Angela Aki	手紙 ～拝啓十五の君へ～	chill 13
HARCO	世界でいちばん頑張ってる君に	chill 13
FUNKY MONKEY BABYS	あとひとつ	chill 13
Field of view	Dreams	chill 14
Field of view	この街で君と暮らしたい	chill 14
19	あの紙ヒコーキくもり空わって	chill 14
Ayaka Hirahara	jupiter	chill 15
HY	366 日	chill 15
Satoshi Oono	Take me faraway	chill 15
Honey L Days	まなざし	chill 16
RADWIMPS	ふたりごと	chill 16
AKB48	会いたかった	chill 16
JAMProject	Name ～君の名は～	chill 17
JAMProject	未来への咆哮	chill 17
JAMProject	GONG	chill 17
Mika Nakashima	雪の華	chill 18
Ayaka Hirahara	jupiter	chill 18
MISIA	Everything	chill 18
Ayaka	夢を味方に	chill 19
いきものがかり	ふたり (Album version)	chill 19

Kou Shibasaki	Nervous	chill 19
BUMP OF CHICKEN	stage of the ground	chill 20
JAMProject	Crest of 'Z's'	chill 20
BUMP OF CHICKEN	sailing day	chill 20
Ringo Sheena	Spica	chill 21
ASIAN KUNG-FU GENERATION	ソラニン	chill 21
Perfume	Dream Fighter	chill 21
東方神起	明日は来るから	chill 22
東方神起	Love in the Ice	chill 22
FTISLAND	Treasure	chill 22
QURULI	春風	chill 23
RC SUCCESSION	君が僕を知ってる	chill 23
Ringo Sheena	Morphine	chill 23
FUNKY MONKEY BABYS	あとひとつ	chill 24
EXILE	道	chill 24
EXILE	Lovers Again	chill 24
RADWIMPS	有心論	chill 25
B'z	今夜月の見える丘に	chill 25
Mai Kuraki	Time after time	chill 25
HY	366 日	chill 26
Superfly	愛をこめて花束を	chill 26
ゆず	栄光の架橋	chill 26
Angela Aki	Kiss Me Good-Bye	chill 27
Angela Aki	This Love	chill 27
Angela Aki	愛の季節	chill 27
MONGOL800	face to face	chill 28
MONGOL800	琉球愛歌	chill 28
ROAD OF MAJOR	雑草	chill 28
Yoshiki Fukuyama	真赤な誓い	chill 29
supercell	君の知らない物語	chill 29
Aya Hirano	God knows	chill 29
いきものがかり	青春ライン	chill 30

Angela Aki	手紙 ～拝啓十五の君へ～	chill 30
BUMP OF CHICKEN	Karman	chill 30
UVERworld	心とココロ	chill 31
RADWIMPS	Ground zero	chill 31
RADWIMPS	Order made	chill 31
Mika Nakashima	Life	chill 32
Mr. Children	花の匂い	chill 32
東方神起	why	chill 32
RADWIMPS	me me she	tear 1
BUMP OF CHICKEN	飴玉の唄	tear 1
BUMP OF CHICKEN	才悩人応援歌	tear 1
FUNKY MONKEY BABYS	Hero	tear 2
FUNKY MONKEY BABYS	もう君がいない	tear 2
FUNKY MONKEY BABYS	あとひとつ	tear 2
BUMP OF CHICKEN	K	tear 3
Lia	夏影	tear 3
BUMP OF CHICKEN	Guild	tear 3
Mr. Children	HANABI	tear 4
YUI	GLORIA	tear 4
aiko	キラキラ	tear 4
RADWIMPS	狭心症	tear 5
Kazumasa Oda	言葉にできない	tear 5
Sakanaction	目が明く藍色	tear 5
Maiko Fujita	運命の人	tear 6
YUI	CHE.R.RY	tear 6
ALI PROJECT	暗黒サイケデリック	tear 6
PornoGraffitti	Winding Road	tear 7
PornoGraffitti	あなたがここにいたら	tear 7
レミオロメン	3 月 9 日	tear 7
コブクロ	赤い糸	tear 8
Mao Abe	いつの日も	tear 8
Motohiro Hata	アイ	tear 8

EXILE	願い (Album version)	tear 9
EXILE	愛すべき未来へ	tear 9
AI	Story	tear 9
コブクロ	蕾	tear 10
Motohiro Hata	アイ	tear 10
Chihiro Onitsuka	眩暈	tear 10
平川地一丁目	ふり向けば戻り道	tear 11
SPLAY	冬の空	tear 11
Hanako Oku	あなたに好きと言われたい	tear 11
Toshihire Baba	スタートライン ～新しい風	tear 12
HY	NAO	tear 12
コブクロ	赤い糸	tear 12
RADWIMPS	もしも	tear 13
DREAMS COME TRUE	何度でも	tear 13
HY	Song for	tear 13
Angela Aki	手紙 ～拝啓十五の君へ～	tear 14
Angela Aki	This Love	tear 14
いきものがかり	ありがとう	tear 14
Every Little Thing	Graceful World	tear 15
Janne Da Arc	still	tear 15
Do As Infinity	Field of dreams	tear 15
いきものがかり	君と歩いた季節	tear 16
CARAMEL PEPPERS	遠距離恋愛 SONG	tear 16
コブクロ	虹	tear 16
いきものがかり	ありがとう	tear 17
YUI	CHE.R.RY	tear 17
FUNKY MONKEY BABYS	告白	tear 17
GReeeeN	またね。	tear 18
Motohiro Hata	朝が来る前に	tear 18
BUMP OF CHICKEN	Guild	tear 18
Mr. Children	旅立ちの唄	tear 19
いきものがかり	今走り出せば	tear 19

DREAMS COME TURE	未来予想図２	tear 19
FUNKY MONKEY BABYS	希望の唄	tear 20
GReeeeN	キセキ	tear 20
世界の終わり	虹色の戦争	tear 20
Surface	夢の続きへ	tear 21
FLOW	Sign	tear 21
Siori Takei	桜色	tear 21
スキマスイッチ	Ice cream syndrome	tear 22
AKB48	桜の木になろう	tear 22
Kisuke Kuwata	明日晴れるかな	tear 22
Ulfuls	暴れだす	tear 23
Mr. Children	くるみ -for the Film 幸福な食卓-	tear 23
Mr. Children	CANDY	tear 23
aiko	キラキラ	tear 24
Tee	Baby, I love you	tear 24
RADWINPS	me me she	tear 24
B'z	いつかのメリークリスマス	tear 25
いきものがかり	帰りたくなったよ	tear 25
Kazumasa Oda	ラブストーリーは突然に	tear 25
Ai Otuka	Pocket	tear 26
SEAMO	マタアイマショウ	tear 26
Ai Otuka	Planetarium	tear 26
MISIA	逢いたくていま	tear 27
JUJU	やさしさで溢れるように	tear 27
JUJU	明日がくるなら	tear 27
Cocco	もくまおう	tear 28
Cocco	ジュゴンの見える丘	tear 28
東京事変	落日 2	tear 28
ゆず	栄光の架橋	tear 29
Yui Aragaki	赤い糸	tear 29
Aqua Timez	千の夜をこえて	tear 29
Shion Tuji	skycord 〜大人になる君へ〜	tear 30

Mai Kuraki	Secret of my heart	tear 30
コブクロ	風	tear 30
MINMI	imagine	tear 31
SunMin	Love You	tear 31
Daichi Miura	Knock Knock Knock	tear 31
JUNSU from 東方神起	Rainy Night	tear 32
FUNKY MONKEY BABYS	もう君がいない	tear 32
JUNSU/JEJUNG/YUCHUN	いつだって君に	tear 32
aiko	ヒカリ	tear 33
Every Little Thing	また あした	tear 33
Kou Shibasaki	ひと恋めぐり	tear 33
UVERworld	君の好きなうた	tear 34
Hikaru Utada	Prisoner of Love	tear 34
EXILE	Lovers Again	tear 34

Musical features

In order to know psychoacoustic features of chills and tears songs, we analyse stimuli by MIR (Music Information Retrieval) toolbox 1.6.1[1]. Table S2 shows musical features of above chills and tears songs as a function of group and the results of the t-tests for the chills and tears groups. Because some participants selected same song, we excluded duplicative 28 songs from the analysis. Chills songs were significantly higher event density, tempo, zerocross, brightness, spectral central, and spectral entropy than tears songs. Whereas tears songs were significantly higher spectral skewness and spectral kurtosis than chills songs. The results indicate that chills songs were characterized by high speed, many notes, and including higher spectral frequency. Tears song were characterized by stable low frequency timbre. This could reflect to subjective emotional responses.

Table S2. Musical features for chills and tears elicited songs

Acoustic features	Mean (SD)		t-test		
	Chill	Tear	*T*	*p*	
Dynamics					
Rms	0.105 (0.003)	0.106 (0.003)	-1.91	.06	
Low energy	0.461 (0.048)	0.464 (0.044)	-0.44	.66	
Event density	2.987 (0.786)	2.629 (0.755)	2.73	.007	**
Rhythm					
Tempo	145.6 (28.3)	137.2 (27.1)	2.14	.03	*
Pulse clarity	0.535 (0.138)	0.532 (0.134)	-0.38	.70	
Timbre					
Zerocross	1479 (348)	1317 (292)	2.89	.004	**
Rolloff	8138 (1237)	7831 (1287)	1.54	.12	
Brightness	0.565 (0.065)	0.540 (0.061)	2.24	.03	*
Spectral central	3683 (505)	3515 (474)	2.04	.04	*
Spectral spread	4212 (392)	4162 (396)	0.82	.41	
Spectral skewness	1.608 (0.262)	1.702 (0.270)	-2.14	.03	*
Spectral kurtosis	5.233 (1.065)	5.607 (1.226)	-1.99	.05	*
Spectral flatness	0.294 (0.059)	0.283 (0.057)	1.05	.29	
Spectral entropy	0.930 (0.010)	0.925 (0.010)	2.83	.005	**

Pitch				
Pitch (mono)	643.4 (367.7)	608.7 (351.7)	0.66	.66
Inharmonicity	0.483 (0.008)	0.482 (0.007)	0.81	.42

1. Lartillot, O., & Toiviainen, P. A matlab toolbox for musical feature extraction from audio. *International Conference on Digital Audio Effects*. Bordeaux. (2007).
